# A rare case report: The impact of *Mycobacterium colombiense* localized infections in a person living with HIV/AIDS

**DOI:** 10.3389/fimmu.2026.1863502

**Published:** 2026-06-30

**Authors:** Lurun Wang, Wenbing Lai, Yuze Liu, Jinwen Huang, Haixia Jing, Xianhui Wang

**Affiliations:** 1 Department of Dermatology, Taihe Hospital, Biomedical Research Institute, Hubei Key Laboratory of Embryonic Stem Cell Research, Hubei University of Medicine, Shiyan, China; 2 Department of Dermatology, The First Affiliated Hospital of Fujian Medical University, Fuzhou, China

**Keywords:** case report, HIV/AIDS, immunocompromised individuals, molecular diagnostics, *Mycobacterium colombiense*, opportunistic infections, skin involvement

## Abstract

Individuals living with HIV/AIDS may be more susceptible to various opportunistic infections due to an immunocompromised status, which can result in diverse clinical manifestations. Among potential pathogens, *Mycobacterium colombiense*—a slow-growing bacterium—could pose a significant threat to such patients. Here, we present the case of a 46-year-old woman with HIV/AIDS who developed an initial dark red plaque that gradually expanded, accompanied by persistent sinus tracts and suppuration. Interestingly, the patient reported no pain or itching. Further examination revealed no evidence of disseminated infection throughout the body. Skin biopsy and histopathological examination, acid-fast staining (AFS), bacterial culture, and molecular diagnostics collectively confirmed the presence of *Mycobacterium colombiense* infection. Treatment with a regimen comprising clarithromycin (500 mg/day), ethambutol (1000 mg/day), and moxifloxacin (400 mg/day), in conjunction with complete surgical excision of the lesion, was administered. During follow-up, no recurrence of the rash has been observed. In conclusion, this case serves as a reminder for clinicians to utilize a multi-faceted diagnostic strategy to identify and manage rare pathogens that may otherwise be overlooked, thereby improving patient outcomes.

## Background

*Mycobacterium colombiense*, a member of the *Mycobacterium avium* complex (MAC), was previously categorized as a rare species of nontuberculous mycobacterium (NTM) (1). It operates as an opportunistic pathogen with a slow growth pattern, typically affecting individuals with immunosuppression (2). Prompt diagnosis can pose challenges due to difficulties in culturing the organism, potentially resulting in delayed diagnoses and deterioration of the clinical condition (2). Here, we present a rare case of an immunosuppressed individual who developed a skin-originating infection with atypical cutaneous lesions. The pathogen was identified as *M. colombiense* following detailed clinical history, histopathological examination, tissue culture, immunohistochemistry and molecular identification.

## Case presentation

Clinical presentation: A 46-year-old woman had an erythematous plaque and sinus tract on the medial side of her left calf, which appeared 1.5 years ago. Three years before this presentation, she had been diagnosed with HIV/AIDS while being treated for cryptococcal meningitis at a specialized Pulmonary Hospital. During that hospitalization, she received amphotericin B for 4 weeks. Post-discharge, she was initiated on tenofovir disoproxil fumarate, lamivudine, and efavirenz. Following achievement of HIV viral load suppression, she was transitioned to Biktarvy for long-term maintenance. During this treatment, her CD4^+^ counts increased from 57 to 168 cells/mm^3^. However, a dark red papule emerged on the left calf, progressively enlarging and accompanied by a persistent sinus and continuous purulent discharge. Notably, the patch was non-pruritic and non-painful, and did not impede mobility. She denied leg trauma, high-risk sexual behavior, and occupational exposure.

**Figure 1:**
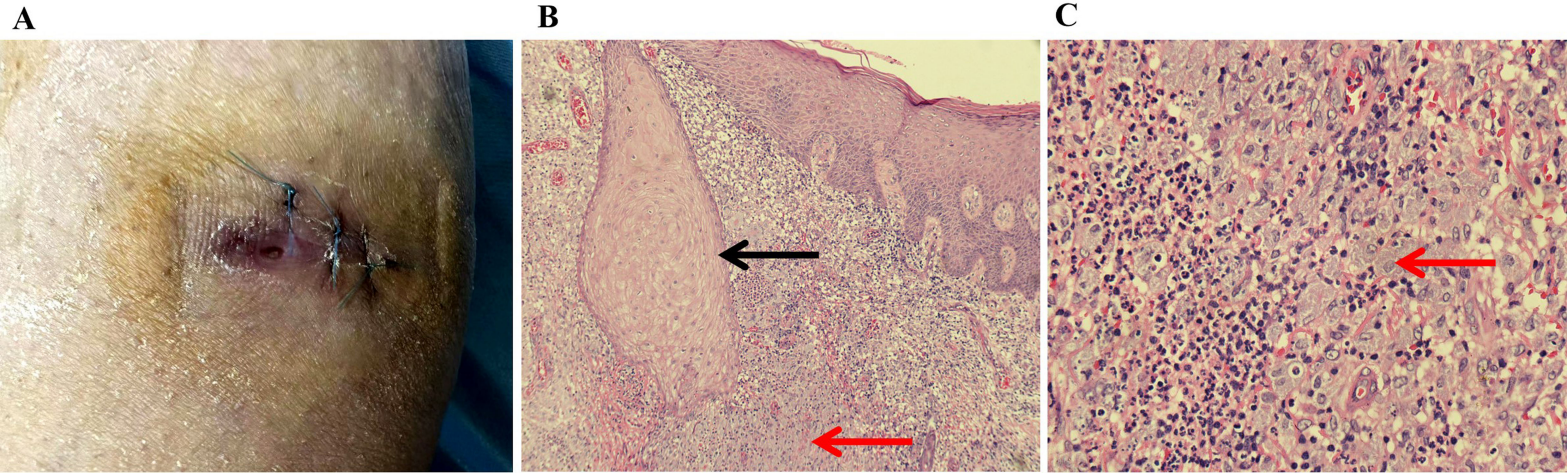
Clinical manifestations. **(A)** Medial side left calf, 0.6-3.0 cm in diameter, with a dark red patch and sinus. **(B, C)** (black arrow) Pseudoepitheliomatous hyperplasia; (red arrow) granuloma inflammation with multinucleated giant cells/histiocytic infiltration (H&E, B: ×100; C: ×200).

**Figure 2:**
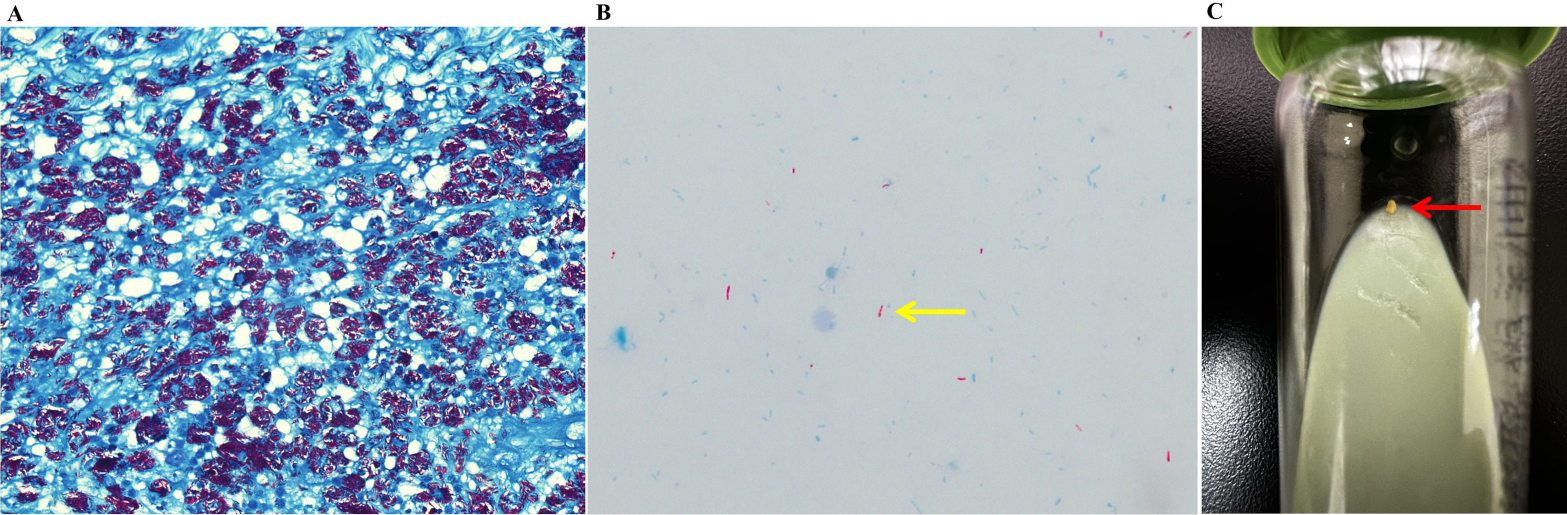
Positive identification of acid-fast bacilli by Ziehl-Neelsen Staining. **(A)** Tissue samples. **(B)** (yellow arrow) In the tissue fluid of skin lesion. **(C)** (red arrow) Smooth, creamy, yolk yellow colonies grew after 3 weeks of incubation (37 °C) on Löwenstei-Jensen medium.

Through systematic physical examination, no abnormalities were found in the heart, lungs, or abdomen, and no obvious swelling was observed in the lymph nodes throughout the body. Upon admission, routine medical examinations, including blood tests, liver function tests, kidney function tests and chest CT examinations, were conducted, and no other abnormalities were found. CD4^+^ cell counts were 153 cells/mm^3^. A skin biopsy of the plaque on the patient’s left leg revealed erosion of the epidermis with pseudoepitheliomatous hyperplasia. In the deep dermis, a non-caseating granulomas was observed, characterized by infiltrated histiocytes, neutrophils, lymphocytes, plasma cells, and blood vessel hyperplasia (**Figure 1**). Additionally, tissue samples and secretions from the sinus tract underwent AFS, cultures, and histology. After 3 weeks of incubation at 37 °C on Löwenstein–Jensen (L-J) medium, moderate growth of smooth, creamy, yolk-yellow colonies was observed (**Figure 2**). Fungal and other standard bacterial cultures were negative.

For specific etiologic diagnosis establishment, genomic DNA was extracted from cells utilizing a QIAamp DNA Mini kit (Qiagen, U.S.A) following the manufacturer’s protocol. PCR and sequencing targeted parts of the *16S rDNA*, *hsp65*, and *rpoB* genes, using universal primers (**Supplementary Table 1**). The sequencing was analyzed with an ABI 3730 Genetic Analyzer (Sang Biotech, Shanghai). Comparisons with the NCBI (https://www.ncbi.nlm.nih.gov/) database showed 98.62% homology with *Mycobacterium* sp. strain MYC012 (GenBank number: MK890456.1) for the *16S rDNA*, 100% similarity with *M. colombiense* clone N45 (GenBank number: OQ658444.1) for the *hsp65*, and 99% homology with *M. colombiense* DSM 45105 (GenBank number: HQ450849.1) for the *rpoB*.

**Figure 3:**
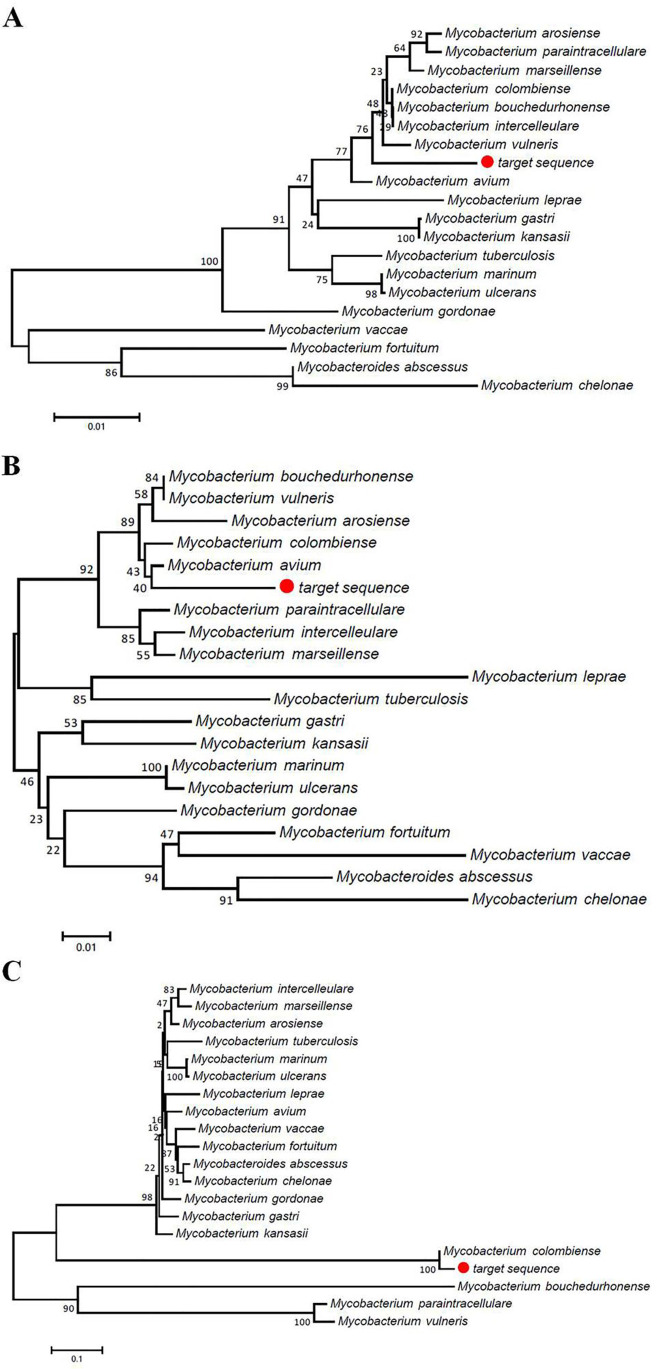
Phylogenetic analysis of *Mycobacterium* species. A concatenated phylogenetic tree, constructed using the neighbor-joining method with Kimura’s two-parameter distance correction, illustrates the evolutionary relationships of the target sequence with reference *Mycobacterium* species based on **(A)** *16S rDNA*, **(B)** *hsp65*, and **(C)** *rpoB* gene.

Further, multiple sequence alignment and phylogenetic tree construction were done using the neighbor-joining plot option of the ClustalW for the *16S rDNA*, *hsp65*, and *rpoB* genes. The trees were validated with 1, 000 bootstrap replicates (3), using *M. tuberculosis* and *M. leprae* as outgroups. The *16S rDNA* sequence analysis showed the species clustering with MAC (**Figure 3A**). The *hsp65* phylogenetic tree revealed the target sequence’s close relation to *M. avium* and *M. colombiense* (**Figure 3B**). The *rpoB*-based phylogenetic analysis confirmed the target sequence’s clustering with *M. colombiense* (**Figure 3C**). These results align with our sequencing findings.

**Figure 4:**
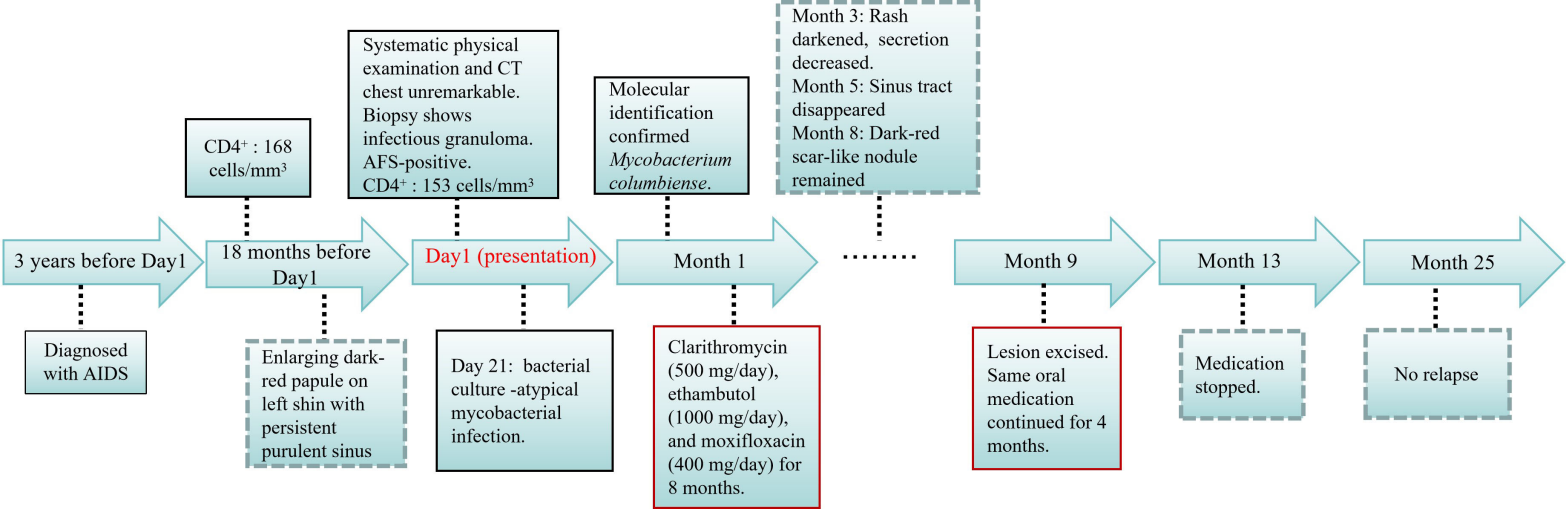
Timeline of key clinical events. The patient was diagnosed with AIDS 3 years before Day 1, and a dark-red papule emerged 18 months before Day 1. On Day 1, biopsy showed infectious granuloma with AFS-positive (CD4^+^ 153 cells/mm³). *M. colombiense* was confirmed on Day 21. Antimicrobial therapy (clarithromycin, ethambutol, moxifloxacin) was administered for 8 months, with lesion excision at Month 9 and postoperative medication continued to Month 13. Follow-up: 12 months after stopping treatment (Month 25), no relapse. Day 1: day of clinical presentation.

In the context of our case, given the possibility of drug interactions between tenofovir, which the individual was taking, and rifampicin, we chose an oral regimen comprising clarithromycin (500 mg/day), ethambutol (1000 mg/day), and moxifloxacin (400 mg/day) for 8 months. The treatment resulted in a substantial lightening of the rash color and a significant reduction in its size. Subsequently, the lesion was completely excised through surgical intervention. The same oral antimicrobial regimen was continued for an additional 4 months postoperatively. To date, no recurrence has been observed during follow-up. The timeline illustrating the entire treatment process of the patient is presented in **Figure 4**.

## Discussion and conclusions

M. *colombiense* was identified in sputum and blood samples from six Colombian patients infected with HIV in 2006 (4). As part of the MAC, it typically causes disseminated infections involving multiple organs, with fever, lymphadenopathy, ostealgia, and skin lesions being the prominent symptoms (5). However, in our case, the infection remained localized to the skin despite the patient being in the advanced stage of HIV infection with a CD4^+^ count of 153 cells/mm^3^ at presentation. Although this count indicated immunosuppression, it was above the critical threshold of 100 cells/mm^3^ below which patients are more susceptible to disseminated MAC infections (6), potentially preserving sufficient immune function for local containment. Clinically, the patient presented with an isolated dark-red, non-pruritic, non-painful plaque and sinus tract on the left calf, without fever, lymphadenopathy, or other systemic manifestations. Normal chest CT and negative blood cultures at presentation further excluded disseminated disease. Furthermore, her regular antiretroviral therapy had raised her CD4^+^ count from 57 to 168 cells/mm^3^, suggesting partial immune restoration that may have contributed to preventing systemic dissemination.

Definitive diagnosis necessitates histopathological examination and the isolation of the microorganism from infected tissue (7). In this instance, a biopsy was taken, and pathological findings revealed pseudoepitheliomatous hyperplasia and non-caseating granulomas composed of numerous lymphocytes, histiocytes, and plasma cells. It is noteworthy that such aberrant symptoms can also manifest in other types of infectious diseases. For example, cat-scratch disease, tularemia, lymphogranuloma venereum, and sporotrichosis all have similar pathological manifestations and their own unique clinical features (8–11).

Cat-scratch disease usually follows cat scratches or bites, marked by enlarged lymph nodes that mostly resolve on their own (12). Ulceroglandular tularemia arises from contact with infected animals or tick bites, causing skin lesions like papules and vesicles, along with systemic symptoms such as headache and fever (9). Lymphogranuloma venereum, caused by Chlamydia trachomatis, features purulent inguinal lymphadenitis and systemic symptoms like fever, distinct from other diseases (13). Sporotrichosis shows as dark red, painless nodules along lymphatics, with cigar-shaped yeasts in tissues and white colonies in culture (11). However, considering the patient’s medical history, epidemiological background, lifestyle, positive results from fungal fluorescence staining, and AFS, we suspected an infection with atypical mycobacteria. Subsequently, upon culturing the bacteria, the colony morphology corresponded to that of slowly growing MAC species observed in previous studies (14). These findings align with common characteristics of atypical mycobacteria (14). To ascertain a more precise species categorization, molecular biological techniques were employed. For more accurate species identification, molecular biological methods, including genetic sequencing targeting the *16S rRNA*, *hsp65*, and *rpoB* genes, were employed.

Genetic sequencing targeting genes such as *16S rRNA (*15), *hsp65 (*16), *rpoB (*17), and the *16S-23S rRNA* internal transcribed spacer (18), or a combination of these genes in a multigene approach, has proven necessary for precise species identification. Traditional methods such as MALDI-TOF MS often cannot reliably differentiate MAC species due to their high phenotypic similarity (14). In this study, we employed a multigene sequencing strategy utilizing *16S rDNA*, *hsp65*, and *rpoB* genes to mitigate misidentification resulting from incomplete shared data or data errors in a single sequence. Analysis of sequence similarity and phylogeny indicated that the causative agent of the infection was *M. colombiense*. Therefore, accurate identification of the etiological agent is crucial for the diagnosis and management of infectious diseases, as well as for outbreak detection.

The latest MAC-PD treatment guidelines recommend combined antibiotic therapy comprising clarithromycin, moxifloxacin, rifabutin, amikacin, and ciprofloxacin as effective against *M. avium* complex organisms (19). For *M. colombiense* specifically, recent case reports describe similar treatment regimens with favorable outcomes (20, 21). Minimum inhibitory concentrations for this organism are consistent with closely related MAC species (22). The most recommended treatment for MAC involves a combination of antibiotics for a period of 6–12 months or longer (23). Clinical therapy should be initiated for individuals with HIV/AIDS co-infected with *M. colombiense* until their immune function has recovered, which may extend to at least a year or even for a lifetime. In our case, we opted for an oral regimen of clarithromycin (500 mg/day), ethambutol (1000 mg/day), and moxifloxacin (400 mg/day) for 8 months with significant improvement. The lesion was then completely excised surgically, after which the same oral antimicrobial regimen was continued. To date, no recurrence has been observed during follow-up.

To the best of our knowledge, reports of *M. colombiense* infection are uncommon, leading to a lack of awareness among clinicians. Most of the commercial labs only give the identification of the organism as MAC and not the actual species name. An individual infected with *M. colombiense* may present with disseminated infection affecting multiple organs. This report describes a case of *M. colombiense* infection confirmed by microbiological and molecular methods in a person with HIV/AIDS who presented exclusively with cutaneous manifestations. This case contributes to the limited literature on this rare clinical presentation.

Our report has several limitations. First, although *M. colombiense* infection in immunocompromised hosts often presents with multi-organ involvement, the absence of systemic symptoms at presentation prompted a limited, skin-focused evaluation rather than a comprehensive organ-directed work-up. Second, antimicrobial susceptibility testing was not performed, depriving us of data that could have refined drug selection. Finally, the short follow-up period precluded a definitive assessment of long-term treatment efficacy.

In conclusion, this case report highlights the rare occurrence of localized *M. colombiense* infection in a patient living with HIV/AIDS, emphasizing the importance of comprehensive diagnostic approaches and tailored treatment strategies in managing opportunistic infections in immunocompromised individuals. Given its potential to cause atypical cutaneous lesions, *M. colombiense* should be included in the differential diagnosis of localized skin infections in immunocompromised patients, even in the absence of disseminated disease. Accurate diagnosis and treatment efficacy of *M. colombiense* require an integrated approach, combining detailed clinical history, histopathological examination, immunohistochemistry, tissue culture, and molecular biology techniques. This case serves as a reminder for clinicians to utilize a multi-faceted diagnostic strategy to identify and manage rare pathogens that may otherwise be overlooked, thereby improving patient outcomes.

## Data Availability

The datasets generated and/or analyzed during the current study are available in the GenBank repository (Accession Numbers PX519014, PX549191, and PX549192). Further inquiries can be directed to the corresponding author.
