# Supplementary material for: A rare case report: The impact of Mycobacterium colombiense localized infections in a person living with HIV/AIDS
Source: Front Immunol. 2026 Jun 30;17:1863502. doi: 10.3389/fimmu.2026.1863502 (PMC13364589; doi:10.3389/fimmu.2026.1863502)
Supplement: Supplementary file 1 [file Table1.docx]

Supplementary table. 1 Three pairs of universal primers were utilized for PCR amplification.

| Primer name | Forward | Reverse |
| --- | --- | --- |
| *16S rDNA* | CACATGCAAGTCGAACGGAAAGG | GCCCGTATCGCCCGCACGCT |
| *hsp65* | ACCAACGATGGTGTGTCCAT | CTTGTCGAACCGCATACCCT |
| *rpoB* | GACGACATCGACCACTTCGG | GGGGTCTCGATCGGGCACAT |
